# The Application of Molecular Techniques to Improve the Classification of Limoniidae

**DOI:** 10.3390/insects17080805

**Published:** 2026-08-03

**Authors:** Pasquale Ciliberti, Sigitas Podėnas, Virginjia Podėnienė, Jekaterina Havelka

**Affiliations:** 1 State Scientific Research Institute Nature Research Centre, Akademijos Str. 2, LT-08412 Vilnius, Lithuania; sigitas.podenas@gamtc.lt; 2 Department of Zoology, Institute of Biosciences, Life Sciences Center of Vilnius University, Saulėtekio Av. 7, LT-10257 Vilnius, Lithuania; virginjia.podeniene@gf.vu.lt (V.P.); jekaterina.havelka@gf.vu.lt (J.H.)

**Keywords:** crane flies, Cylindrotomidae, molecular systematics, Pediciidae, morphology, Tipulidae, Tipulomorpha

## Abstract

The family Limoniidae is the most species-rich within the order Diptera. The classification of this family is still unresolved and is based largely on morphological studies and the opinions of a few specialists. The rapid advancement of molecular techniques has created new opportunities to address long-standing systematic challenges. Nevertheless, relatively few studies have used molecular data to refine our understanding of Limoniidae and its relationships with allied lineages. In the present study, we reviewed publications that applied molecular approaches to improve the classification of Limoniidae and clarify its relationships with related groups. Relevant studies were identified through the literature section of the Catalogue of the Craneflies of the World using the keywords “classification”, “systematics”, and “molecular”. We retained only studies that employed molecular data to investigate the classification of Limoniidae, the phylogenetic position of the family within Tipuloidea, and the placement of Tipulomorpha within the dipteran tree. The studies reviewed consistently support the conclusion that Limoniidae is not monophyletic. In contrast, Tipulomorpha is consistently recovered as a monophyletic lineage, although its position within the dipteran tree remains controversial. Consequently, a satisfactory taxonomic framework has yet to be achieved. Recent advances in high-throughput sequencing and the generation of large-scale molecular datasets provide unprecedented opportunities to resolve these systematic uncertainties. We therefore advocate expanding both taxon sampling and the number of genetic markers included in phylogenetic analyses. Ultimately, a coordinated effort among research groups and institutions is likely to represent the most effective strategy for resolving the taxonomy and evolutionary relationships of Limoniidae.

## Simple Summary

Classification is the process of grouping living organisms according to their shared characteristics. It helps scientists understand how species are related through evolution, how they have adapted to different environments, and how to consistently identify and name them. With more than 10,000 described species, Limoniidae, or short-palped crane flies, are the largest group of crane flies. Their classification has traditionally been based on their physical features, but it remains controversial. Despite decades of research, scientists still do not agree on how the different groups within Limoniidae are related to one another or to other crane flies. Recent advances in DNA-based methods have greatly improved our ability to study how species are related. However, only a few studies have used DNA data to investigate the classification of Limoniidae, and their results have often disagreed. In this article, we review these studies and argue that a coordinated collaboration among crane fly researchers is needed to resolve these long-standing questions. We also highlight the potential of phylogenomics, an approach that compares large amounts of DNA from many species, to provide a more stable and reliable classification of Limoniidae and other crane flies.

## 1. Introduction

The family Limoniidae is a lineage of nematocerous flies and represents the most species-rich group within the order Diptera, with nearly 11,000 described species [1]. However, the classification of this lineage and its relationships with other nematocerous flies remain a matter of debate. Within the family, many species exhibit remarkable morphological diversity in both adult and larval stages, making the identification of reliable diagnostic and phylogenetically informative characters particularly challenging [2].

Limoniidae are closely related to a group of flies commonly known as crane flies. Crane flies are easily recognized by their slender bodies and long legs. Currently, four major lineages of crane flies are recognized: Cylindrotomidae or long-bodied crane flies, Limoniidae or short-palped crane flies, Pediciidae or hairy crane flies, and Tipulidae or long-palped crane flies [2]. Few morphological characters distinguish these four lineages in the adult stage. Notable features include a tripartite male aedeagus in Cylindrotomidae and an atrophied vein *R*_1+2_ ending in *R*_3_. Limoniidae are primarily distinguished from other crane flies by their short final palpomere, whereas Tipulidae have a long palpomere. The main distinguishing feature of Pediciidae is the presence of ommatrichia between the eye facets. The higher-level classification of these groups remains unclear, particularly regarding the taxonomic rank that should be assigned to each lineage. Two primary classification systems are commonly used (Table 1). The one-family system groups all four lineages under the family Tipulidae, with Limoniinae treated as a subfamily within this family [3]. The four-family system treats each lineage as a separate family within the superfamily Tipuloidea, which comprises Cylindrotomidae, Limoniidae, Pediciidae and Tipulidae (Table 1; [2,4,5]). At a higher taxonomic level, crane flies are placed within the infraorder Tipulomorpha, which includes the four families of Tipuloidea along with Trichoceridae (winter crane flies), considered the putative sister group of Tipuloidea [6].

The choice between the one-family and four-family systems appears to be largely influenced by the historical and geographical background of the taxonomists involved [7]. Before 1900, authors typically classified crane flies as a single family. However, in the late 19th century, some authors already accepted the partition of the family Tipulidae into two divisions: Tipulidae longipalpi and Tipulidae brevipalpi, based on the length of the terminal palpal segment [8]. In the early 20th century, European entomologists began treating Tipulidae brevipalpi as a separate family, initially called Limnobiidae, based on the genus name *Limnobia* Meigen, 1818 (e.g., [9]) later corrected to Limoniidae, based on the genus name *Limonia* Meigen, 1803. In contrast, Charles P. Alexander’s extensive work in North America maintained the one-family system [2]. With over 1000 published papers and descriptions of more than two-thirds of known crane fly species, Alexander remains the most prolific crane fly specialist to date [1]. His influence led most North American entomologists to adopt the one-family classification. This approach was also prevalent in the United Kingdom, likely due to the influence of another prominent dipterist, Frederick W. Edwards [7]. Meanwhile, in Central and Eastern Europe, taxonomic work by researchers such as Lackschewitz [10], Savchenko [11,12], and Mannheims [13] strongly influenced regional practice, leading to broader acceptance of the multi-family system outside the UK.

The multi-family system adopted by most European workers was later refined by Starý in 1992 [4], who elevated the Pediciinae to the family rank and proposed to split the family Limoniidae in four subfamilies. The four-family system of Tipuloidea proposed by Starý [4] was accepted by most European workers, and we will use it throughout the remainder of this short review.

**Figure 1:**
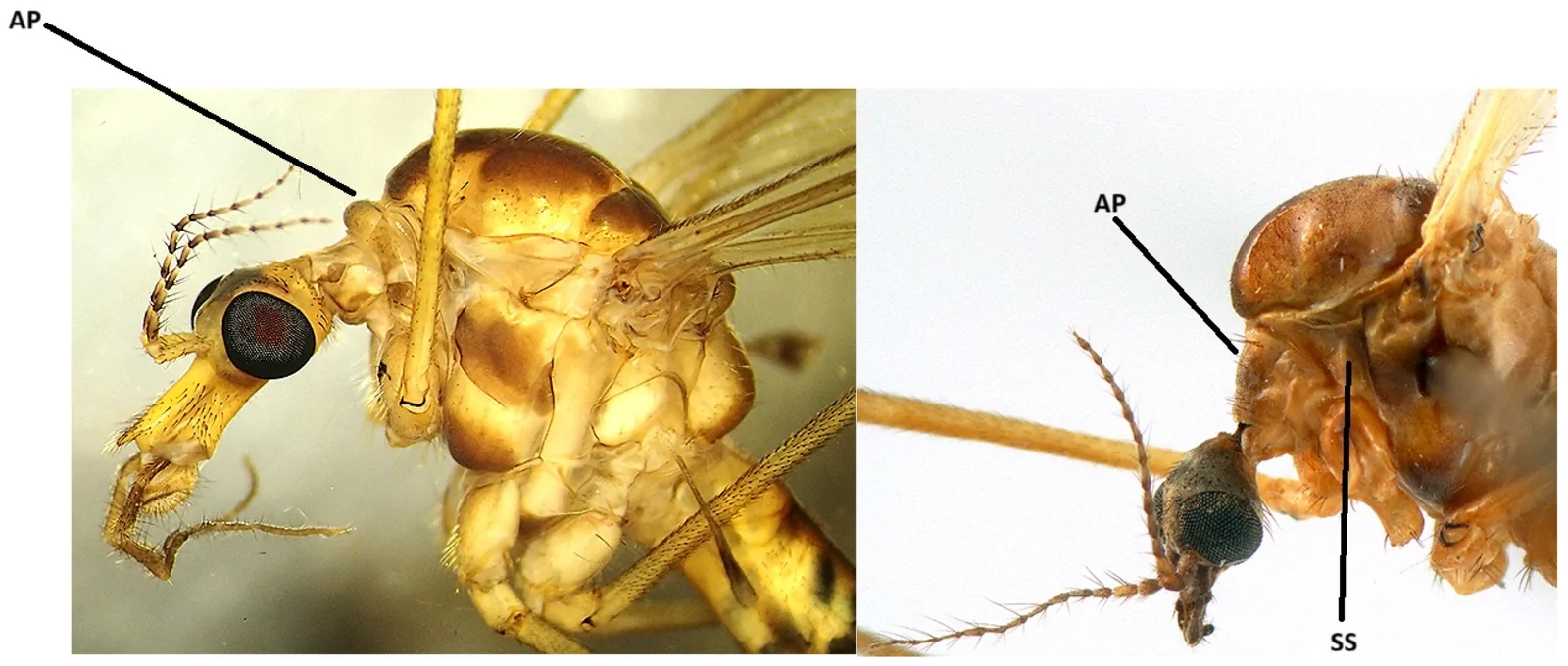
Synapomorphies of Limoniidae according to Starý [4]. (**Right**) *Tipula alpina* Loew, 1873 showing the short and collar-shaped antepronotum. (**Left**) *Limonia taurica* (Strobl, 1895) with the flattened and elongated antepronotum and the subspiracular sclerite. Abbreviations: AP = antepronotum, SS = subspiracular sclerite. Images downloaded from the Catalogue of Craneflies of the World. Photo of *T. alpina* by K.M. Olsen. Photo of *L. taurica* by P. Oosterbroek.

The analysis of Starý [4] is essentially based on his extensive expertise, but remains qualitative and somewhat subjective. While his work examined a range of morphological characters, Starý [4] concluded that 11 of them could support the monophyly of the infraorder Tipulomorpha. In his view, Limoniidae is a monophyletic family defined by two key synapomorphies: (1) an elongated and flattened antepronotum, and (2) the presence of a subspiracular sclerite (Figure 1). The pronotum in Tipulomorpha is divided into an anterior and posterior part, separated by a membranous transnotal suture. In the crane fly lineages other than Limoniidae, the antepronotum is short and collar-shaped, while in Limoniidae it is both elongated and flattened (Figure 1). Additionally, the postpronotum extends downward in a strip-like manner, adjacent to which is a membranous area. In Limoniidae, however, this membranous area is partially replaced by a sclerite, which Starý [4] referred to as the subspiracular sclerite (Figure 1). These morphological traits are absent in the Pediciinae, which led Starý [4] to elevate this subfamily to family rank, separating it from Limoniidae. Furthermore, the Pediciidae are unique in having ommatrichia between the eyes, further justifying their distinction from the Limoniidae. Starý [4] also elevated the genus *Dactylolabis* Osten Sacken, 1860 to subfamily level, primarily based on the presence of fleshy gonostyli, a feature not shared by the other three subfamilies. However, the characters used to define subfamilies within Limoniidae, such as the presence or absence of tibial spurs, the number of radial and medial veins reaching the wing margin, the size of the meron, and the way the subspiracular sclerite is fused with the postpronotum, are inconsistently distributed across the genera. This inconsistency makes the Limoniidae concept proposed by Starý [4] problematic. Before Starý’s classification [4], the monophyly of Limoniidae had already been questioned by morphological studies. For example, Oosterbroek and Theowald [14] analysed 105 characters from crane fly larvae and pupae, and their results suggested that Limoniidae is not monophyletic.

A taxonomic group is monophyletic if it includes the most recent common ancestor and all of its descendants [15]. The analysis of Oosterbroek and Theowald [14] identified three clades: the first comprising Chioneinae and Limnophiliinae; the second containing Pediciidae; and a third consisting of Limoniinae and unplaced genera, alongside a well-supported sister relationship between Cylindrotomidae and Tipulidae.

Ribeiro [16], focusing on crane fly relationships with an emphasis on Limnophiliinae, proposed that this subfamily is the most ancient lineage within the Limoniidae. Although his study confirmed the non-monophyly of the Limoniidae, the analysis, based exclusively on adult male characters, yielded trees with high levels of homoplasy. Ribeiro’s results further identified Pediciidae as the most basal crane fly lineage, while nesting Cylindrotomyidae and Tipulidae within the Limoniidae.

The prevailing view among specialists is that the current classification of Limoniidae is unsatisfactory and likely inconsistent with their evolutionary history [5]. Nevertheless, practical considerations and the absence of a robust alternative hypothesis have led most researchers to continue using Starý’s framework [4].

The rapid advancement of molecular biology, along with bioinformatics have given researchers new and powerful tools to attempt to solve problems in systematics [17].

The few molecular studies employed to improve the classification of Limoniidae have focused on answering two questions: (1) what are the phylogenetic relationships between Limoniidae and the other lineages of the Tipulomorpha, and what is the position of these groups within the dipteran tree? (2) what are the evolutionary relationships among the subfamilies of Limoniidae and between these subfamilies and those of the other tipuloid subfamilies?

In this short review we provide an overview of those studies. The articles reviewed were selected by searching the literature section of the Catalogue of the Craneflies of the World using the keywords “classification”, “systematics” and “molecular”. We retained only articles that focused on the classification of Limoniidae or the phylogenetic position of Limoniidae and Tipulomorpha within the dipteran phylogeny. In total, six studies using molecular approaches to investigate the phylogenetic position of Limoniidae and the Tipulomorpha and eight studies addressing the classification of Limoniidae were included (Table 2 and Table 3).

**Table 1:** Alternative classification schemes of crane flies. Note that before the work of Starý [4] the Pediciinae lineage was mostly treated as a tribe of the Limoniinae subfamily in the one family system and as a subfamily of Limoniidae in the multi-family system. Classification follows Wiegmann et al. [18] at the infraorder level, Starý [4] at the superfamily level, and Alexander [3] at the family level. Note that in the classification of Alexander [3] the name Limnobiinae is used instead of Limoniinae.

Infraorder Tipulomorpha	Superfamily Tipuloidea	Family Tipulidae
Families	Families	Subfamilies
Trichoceridae Cylindrotomidae Limoniidae Pediciidae Tipulidae	Cylindrotomidae Limoniidae Pediciidae Tipulidae	Cylindrotominae Tipulinae Limnobiinae **Tribe** Pediciini

## 2. Classification of Limoniidae Using Molecular Techniques

### 2.1. Position of the Limoniidae and Tipulomorpha Within the Diptera

**Figure 2:**
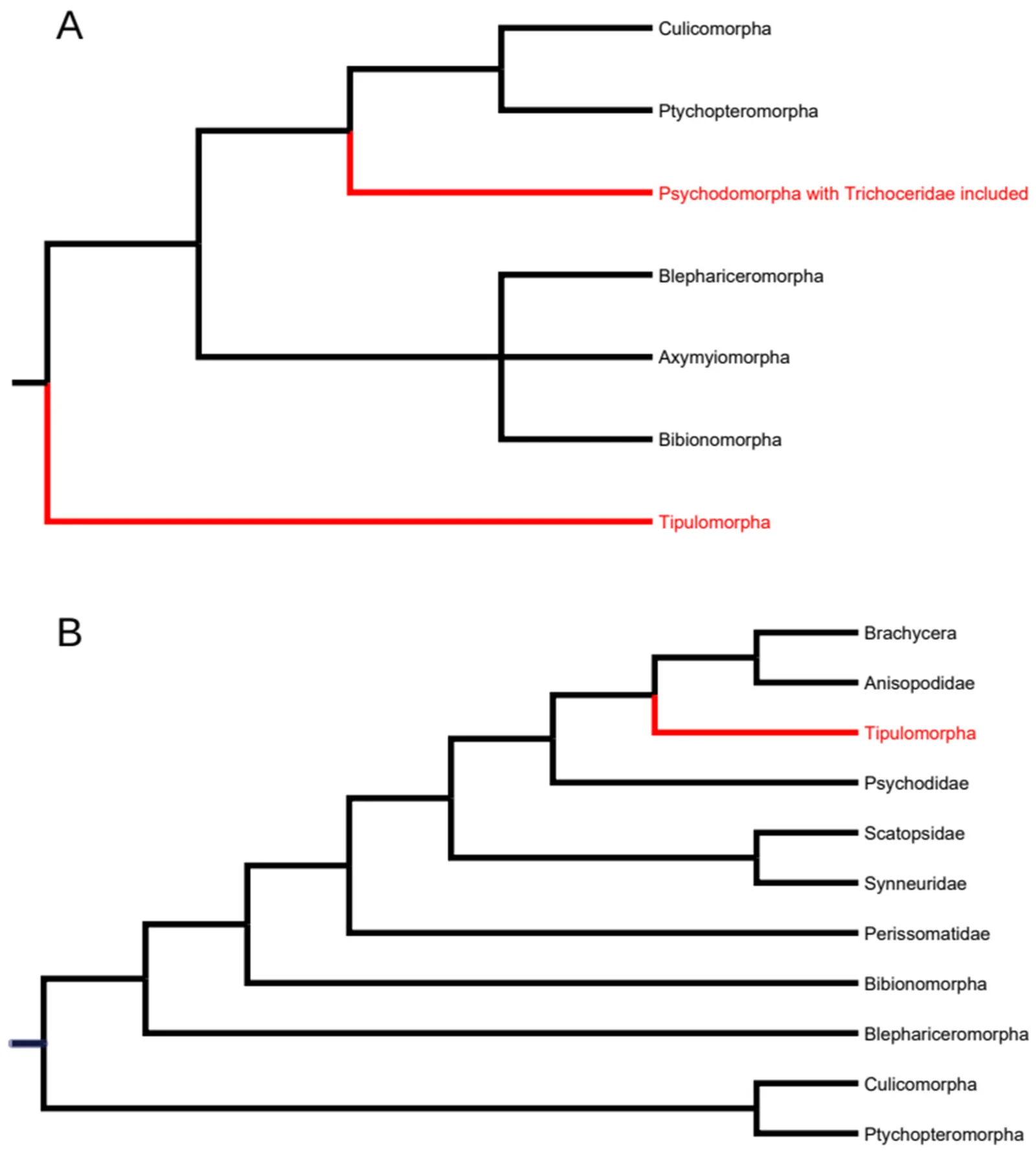
Examples of Diptera phylogenies based on morphological studies showing contrasting hypothesis for the placement of the Tipulomorpha. Notice that in (**A**) the Tipulomorpha is not retrieved as a monophyletic clade. (**A**)—after Wood & Borkent [23]. (**B**)—after Oosterbroek & Courtney [24].

**Figure 3:**
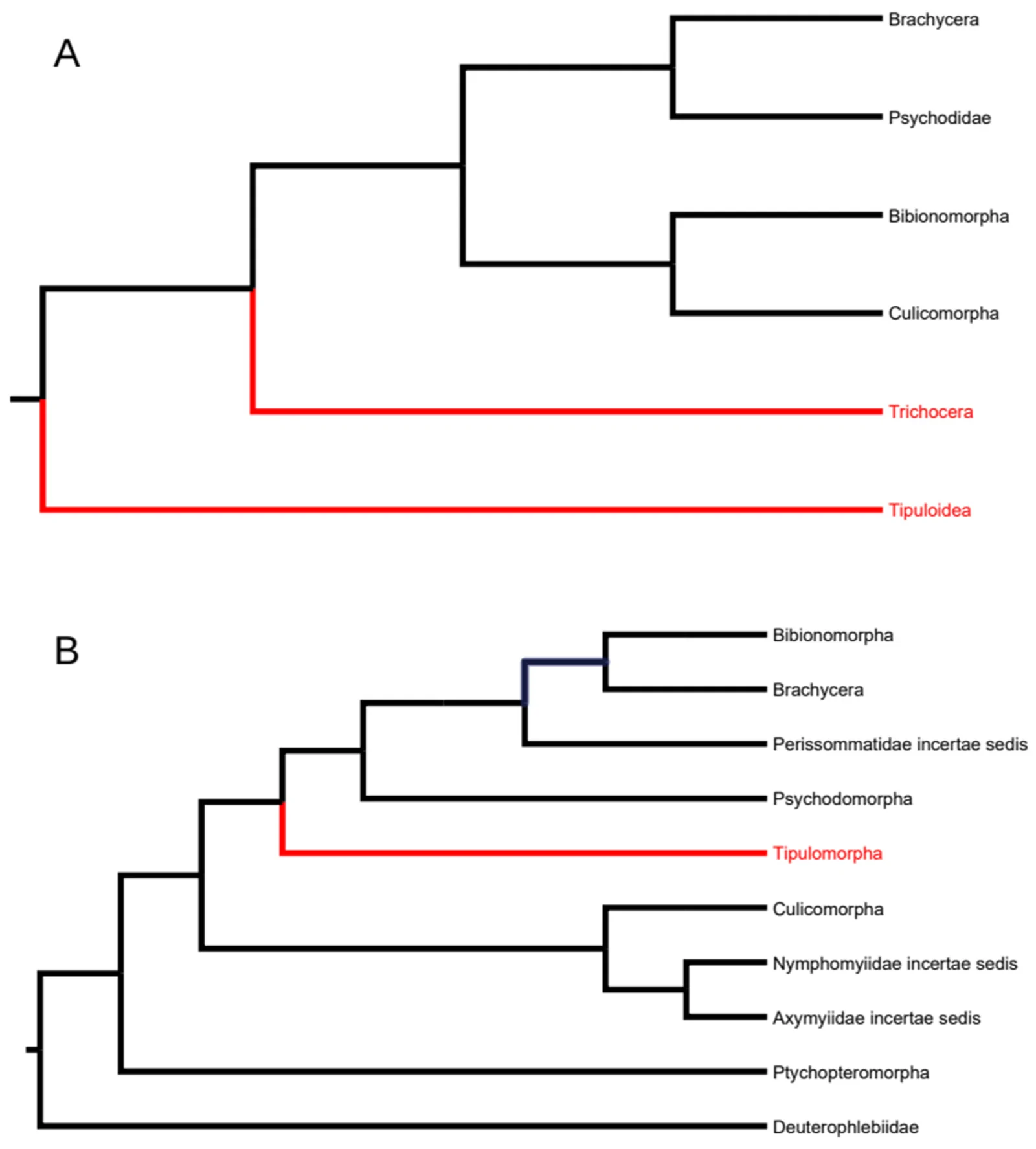
Examples of dipteran phylogenies inferred from molecular studies. Note that in (**A**), the infraorder Tipulomorpha is not recovered as monophyletic. (**A**)—modified from the Maximum Likelihood phylogeny of Friedrich & Tautz [19]. (**B**)—modified from the Bayesian inference phylogeny based on the concatenated dataset of Bertone et al. [25].

The first molecular investigation addressing the position of the Limoniidae and other crane flies within the Diptera tree was conducted by Friedrich and Tautz in 1998 [19]. Although their primary goal was to resolve the phylogeny of the Lower Diptera. Diptera is an order of holometabolous insects traditionally divided into two suborders: the Nematocera, or “thread-horn flies,” characterized by long multi-segmented antennae, and the Brachycera, or “short-horn flies,” characterized by a reduction in the number of flagellomeres [20]. It is widely accepted that the Nematocera, also known as the Lower Diptera, is paraphyletic, whereas the Brachycera form a monophyletic clade [21]. This implies that the taxa treated traditionally as Nematocera do not represent a natural evolutionary group but rather different lineages. Based on fossils specimens from the Triassic and Lower Jurassic, Krzeminski [22] proposed to divide the order Diptera in four suborders. However, this classification was not followed by researchers working with recent fauna. Morphological studies have identified five or more major clades within the Nematocera, commonly referred to as infraorders [23,24] (Figure 2). Amorim and Yeates [21] later proposed treating these infraorders as suborders but because the phylogeny of the Nematocera is still unresolved the rank of infraorder is still preferred. The composition and interrelationships of these infraorders vary across different investigations. Particularly challenging is identifying both the basal lineage of Diptera and the lineage of Lower Diptera from which the Brachycera evolved. Morphological studies have produced conflicting hypotheses regarding the position of crane flies. Limoniidae and other crane fly lineages have been placed by Hennig [6] and Wood & Borkent [23] as the most basal lineage of Diptera (Figure 2A). However, the study by Wood and Borkent [23], based mainly on larval characters, removed Trichoceridae from Tipulomorpha and reassigned it to Psychodomorpha (Figure 2A). Oosterbroek and Courtney [24], in contrast positioned the Tipulomorpha as a more derived group, sister to a clade composed of Anisopodidae and Brachycera (Figure 2B). Friedrich and Tautz [19] revisited these questions using fragments of the 28S rRNA gene. Only 488 base pairs could be confidently aligned, and three phylogenetic reconstruction methods, Maximum Parsimony, Maximum Likelihood, and Neighbor Joining, were applied. Although all analyses recovered Tipuloidea as monophyletic (excluding Trichoceridae), the relative placement of major infraorders varied among trees. Tipuloidea appeared basal under Maximum Likelihood (Figure 3A) but not under Neighbor Joining [19]. The study relied on very limited taxon sampling (16 ingroup and three outgroup species), including only two tipuloid species and a single trichocerid, which likely contributed to the low support values and instability of the inferred relationships. Bertone et al. [25] employed multiple nuclear genes to analyse Lower Diptera relationships (Figure 3B). They sequenced the ribosomal 28S rRNA gene along with the protein-coding genes Carbamoyl-phosphate synthetase 2/Aspartate transcarbamylase/Dihydroorotase (CAD), Triosephosphate isomerase (TPI), and 6-phosphogluconate dehydrogenase (PGD). After alignment and the exclusion of ambiguous sites, 5272 characters remained. Three phylogenetic approaches were used: Maximum Parsimony, Maximum Likelihood, and Bayesian inference.

**Table 2:** Molecular studies investigating the phylogeny of lower Diptera and the placement of Tipulomorpha within the dipteran phylogeny included in this review.

Study	Principal Question Investigated	Number of Ingroup Species	Outgroup Species	Number of Tipulomorpha Species	Study Design	Phylogenetic Tree Construction	Main Conclusions
Friedrich and Tautz [19]	Relationships among Nematocera	16	One Lepidoptera, one Mecoptera and one Siphonaptera species	3	Single-gene used to infer phylogeny	Maximum Likelihood, Maximum Parsinomy, Neighbor Joining	Tipuloidea monophyletic, withouth Trichoceridae
Bertone et al. [25]	Relationships among Nematocera	64 lower diptera, 3 Brachycera	Two Mecoptera and one Siphonaptera species	10	Four genes used to infer phylogeny	Maximum Likelihood, Maximum Parsinomy, Bayesian inference	Tipulomorpha monophyletic, intermediate within the Diptera tree
Wiegmann et al. [18]	Phylogeny of the Diptera	Tier1: 42 taxa, Tier 2: 202 taxa	One Lepidoptera, one Siphonaptera and 4 Mecoptera species	9	Two-tier study: Tier 1: 14 nuclear genes, complete mitochondrial genomes, and 371 morphological characters; Tier 2: five nuclear genes. Both tiers were used to infer phylogenetic relationships.	Maximum likelihood, Bayesian inference	Tipulomorpha monophyletic, near base of the Diptera tree
Zhang et al. [26]	Relationships among Nematocera	29	Two Mecoptera and one Siphonaptera species	9	Two combinations of mitochondrial genome used to infer phylogeny: 9815 bp and 6067 bp with several protein-coding genes excluded in the second dataset.	Bayesian inference	Tipulomorpha monophyletic in both dataset and placed in an intermediate position
Kang et al. [27]	Phylogeny of the Tipulomorpha	17 Lower Diptera, three Brachycera	One Mecoptera and one Siphonaptera species	5	1709 single-copy orthologous genes used to infer phylogeny	Maximum likelihood	Tipulomorpha monophyletic, intermediate within the Diptera tree
Zhang et al. [28]	Relationships among Nematocera	108	Four Mecoptera and Four Siphonaptera species	12	13,513 bp of mitochondrial genome used to infer phylogeny	Bayesian inference	Tipulomorpha monophyletic, positioned near base of Diptera tree

**Table 3:** Molecular studies included in this review that investigated the classification of Limoniidae and the phylogenetic relationships within Tipuloidea.

Study	Principal Question Investigated	Number of Ingroup Species	Outgroup Species	Number of Limoniidae Species	Number of Tipuloidea Excl. Limoniidae	Dataset	Phylogenetic Tree Construction	Main Conclusions
Ahonen [29]	Relationship among subfamilies of the Tipulomorpha	37	Two Trichoceridae species	18	17	Two genes used to infer phylogeny	Maximum Parsinomy, Maximum Likelihood, Neighbor Joining, Bayesian Inference	Pediciidae basal lineage, Limnophilinae paraphyletic, Cylindrotomidae and Tipulidae closely related
Petersen et al. [5]	Higher-level classification of Tipuloidea	45	Two Ptychopteridae and two Trichoceridae species	30	15	Two genes + 100 morphological characters used to infer phylogeny	Maximum Parsinomy, Bayesian Inference	Pediciidae basal lineage of Tipuloidea, Limoniidae paraphyletic, Cylindrotomidae and Tipulidae sister taxa.
Kang et al. [30]	Characterization of mitochondriial genome of. *C. crassipes gracylistila* Phylogeny of Tipuloidea	8	Two Trichoceridae species	4	4	Whole mitochondrial genome	Bayesian inference	Pediciidae basal lineage of Tipuloidea, Limoniidae paraphyletic, Cylindrotomidae and Tipulidae sister taxa
Ren et al. [31]	Characterization of mitochondrial genome of *L. phragmitidis.* Phylogeny of Tipuloidea	8	One Trichoceridae species	4	4	13 protein-coding genes of mitochondrial genome	Bayesian inference	Pediciidae basal lineage of Tipuloidea, Limoniidae paraphyletic, Cylindrotomidae and Tipulidae sister taxa
Ren et al. [32]	Characterization of mitochondrial genome of *N. tenuipes* Phylogeny of Tipuloidea	10	One Anisopodidae and two Trichoceridae species	4	6	13 protein-coding genes and 2 rRNAs of mitochondrial genome	Bayesian inference	Pediciidae basal lineage of Tipuloidea, Limoniidae paraphyletic, Cylindrotomidae and Tipulidae
								sister taxa
Ren et al. [33]	Characterization of mitochondrial genome of *P. brunneinota.* Phylogeny of Tipuloidea	11	One Anisopodidae and two Trichoceridae species	5	6	13 protein-coding genes and 2 rRNAs of mitochondrial genome	Bayesian inference	Pediciidae basal lineage of Tipuloidea, Limoniidae paraphyletic, Cylindrotomidae and Tipulidae sister taxa
Kang et al. [34]	Characterization of mitochondrial genome of the tribe *Elephantomyiini* Phylogeny of Tipuloidea	27	Three Trichoceridae species	12	15	Four different mitochondrial dataset combinations.	Maximum Likelihood, Bayesian inference	Pediciidae basal lineage of Tipuloidea, Limoniidae paraphyletic, Cylindrotomidae and Tipulidae sister taxa
Xu et al. [35]	Characterization of mitochondrial genome of the tribe *Dicranoptychini* Phylogeny of Tipuloidea	17	One Pediciidae species	12	5	Four different mitochondrial dataset combinations.	Maximum Likelihood, Bayesian inference	Limoniidae paraphyletic, Cylindrotomidae and Tipulidae sister taxa

The dataset comprised 64 lower dipteran species and three brachyceran species as the ingroup, two Mecoptera species, and one Siphonaptera species as outgroup.

Single-gene analyses produced incongruent or weakly resolved trees, whereas the combined multi-gene dataset, particularly after excluding third codon positions, yielded better-resolved and largely congruent topologies. All trees supported a monophyletic Tipulomorpha, including Limoniidae, Pediciidae, Cylindrotomidae, Tipulidae, and Trichoceridae. However, Tipulomorpha did not emerge as either basal or highly derived within Diptera (Figure 3B). Most of the phylogenetic signal originated from 28S rRNA rather than the protein-coding genes. Surprisingly, Deuterophlebiidae emerged as the most basal dipteran lineage, a result notable given the family’s numerous autapomorphies [25].

**Figure 4:**
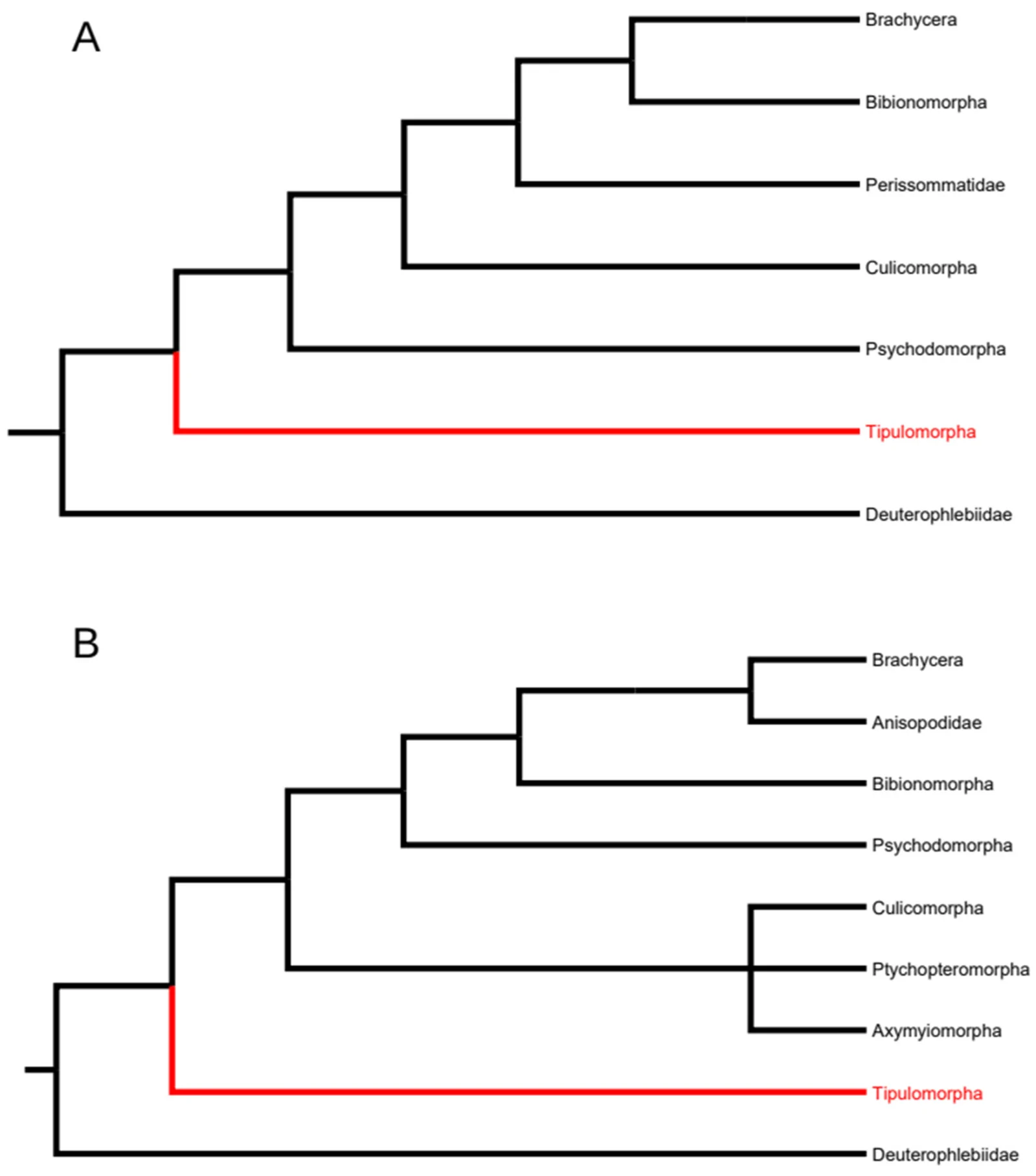
Examples of diptera phylogenies based on molecular studies. (**A**)—after Wiegmann et al. [18]. (**B**)—after Zhang et al. [28].

A study by Wiegmann et al. [18] confirmed the basal placement of Deuterophlebiidae within Diptera (Figure 4A). Their analysis included about 200 species representing 149 of the 157 recognized dipteran families and was conducted in two tiers: tier 1 comprised 42 species, 14 nuclear genes, the complete mitochondrial genome, and 371 morphological characters; tier 2 included 202 taxa and five nuclear genes. Phylogenetic reconstruction using Maximum Likelihood and Bayesian inference confirmed the basal position of Deuterophlebiidae and recovered Tipulomorpha as a monophyletic group placed near the base of the tree (Figure 4A).

Zhang et al. [26] sequenced the complete mitochondrial genome of *Symplecta hybrida* (Meigen, 1804) and obtained five additional nearly complete mitochondrial genomes from other Tipuloidea species. They compared these sequences with mitochondrial genomes available from other dipteran lineages and reconstructed phylogenetic relationships using Bayesian inference. A total of 29 dipteran species were analysed, with two Mecoptera and one Siphonaptera species used as outgroup. Two datasets were assembled. The first comprised the first and second codon positions of the 13 mitochondrial protein-coding genes, the two ribosomal RNA genes, and 19 transfer RNA genes. Because several protein-coding genes proved difficult to align, the authors also analysed a second dataset consisting of the first and second codon positions of five protein-coding genes, the two ribosomal RNA genes, and 19 transfer RNA genes. The two datasets produced conflicting phylogenetic topologies, preventing the authors from drawing firm conclusions regarding the earliest-diverging dipteran lineage or the origin of Brachycera. Nevertheless, both analyses recovered Tipulomorpha as a monophyletic lineage that was neither the earliest-diverging nor the most derived group within Diptera [26]. In 2017, Kang et al. published [27] the most comprehensive phylogenomic study to date investigating the phylogeny of the Tipulomorpha. The authors assembled a dataset of 1709 single-copy orthologs genes derived primarily from transcriptome sequencing. Three transcriptomes were generated specifically for the study, whereas the remaining data were obtained from publicly available resources [27]. Phylogenetic relationships were inferred using Maximum Likelihood. Analyses based on the complete orthologous dataset recovered Culicomorpha as the earliest-diverging lineage within Diptera and Bibionomorpha as the sister group of Brachycera. Tipulomorpha was recovered as a monophyletic lineage occupying an intermediate position within the dipteran phylogeny. However, these conclusions were based on relatively limited taxon sampling, comprising only 20 ingroup species and two outgroup species, including seven Culicomorpha, five Tipulomorpha, two Psychodomorpha, three Bibionomorpha, and three Brachycera species.

More recently, Zhang et al. [28] reconstructed a phylogeny of nematocerous flies based exclusively on mitochondrial genomes, encompassing 108 dipteran species and eight outgroups (Figure 4B). Using Bayesian inference on a concatenated dataset of 13 protein-coding genes, two rRNA genes, and 22 tRNAs (13,513 bp), they again recovered Deuterophlebiidae as the most basal Diptera, albeit with low support. Tipulomorpha emerged as a strongly supported monophyletic clade composed of Cylindrotomidae, Limoniidae, Pediciidae, Tipulidae, and Trichoceridae, and was placed basally relative to most other Diptera (Figure 4B). However, support for the relationships between Tipulomorpha and the remaining Diptera was low [28].

In summary, the placement of crane fly lineages within the Diptera remains a complex issue with variable results across studies. The analyses of Wiegmann et al. [18] and Zhang et al. [28], which included the most comprehensive taxon sampling among the studies reviewed, support Tipulomorpha as an early-diverging, but not the earliest-diverging, lineage within Diptera (Figure 4).

Resolving the evolutionary history of this diverse and species-rich order remains challenging but is essential for understanding broader ecological and evolutionary patterns. In particular, the likely rapid radiation of certain dipteran groups has obscured phylogenetic signals; increased sampling of such lineages (e.g., acalyptrate flies), may help in resolving the placement of Tipulomorpha.

### 2.2. Test of the Monophyly of the Limoniidae and Analysis of Crane Flies’ Relationships

Studies on Limoniidae classification have generally examined relationships among crane fly families or tested the monophyly of existing subfamilies. Limoniidae is currently divided into four subfamilies: Chioneinae, Dactylolabinae, Limnophilinae, and Limoniinae [1]. However, morphological analyses have produced inconsistent results and have failed to robustly support the monophyly of Limoniidae; in particular, the status of Limnophilinae remains problematic [16]. Few molecular analyses have directly evaluated limoniid classification, and both morphological and molecular studies have tended to focus on testing established taxonomic groups rather than proposing new classifications [2].

**Figure 5:**
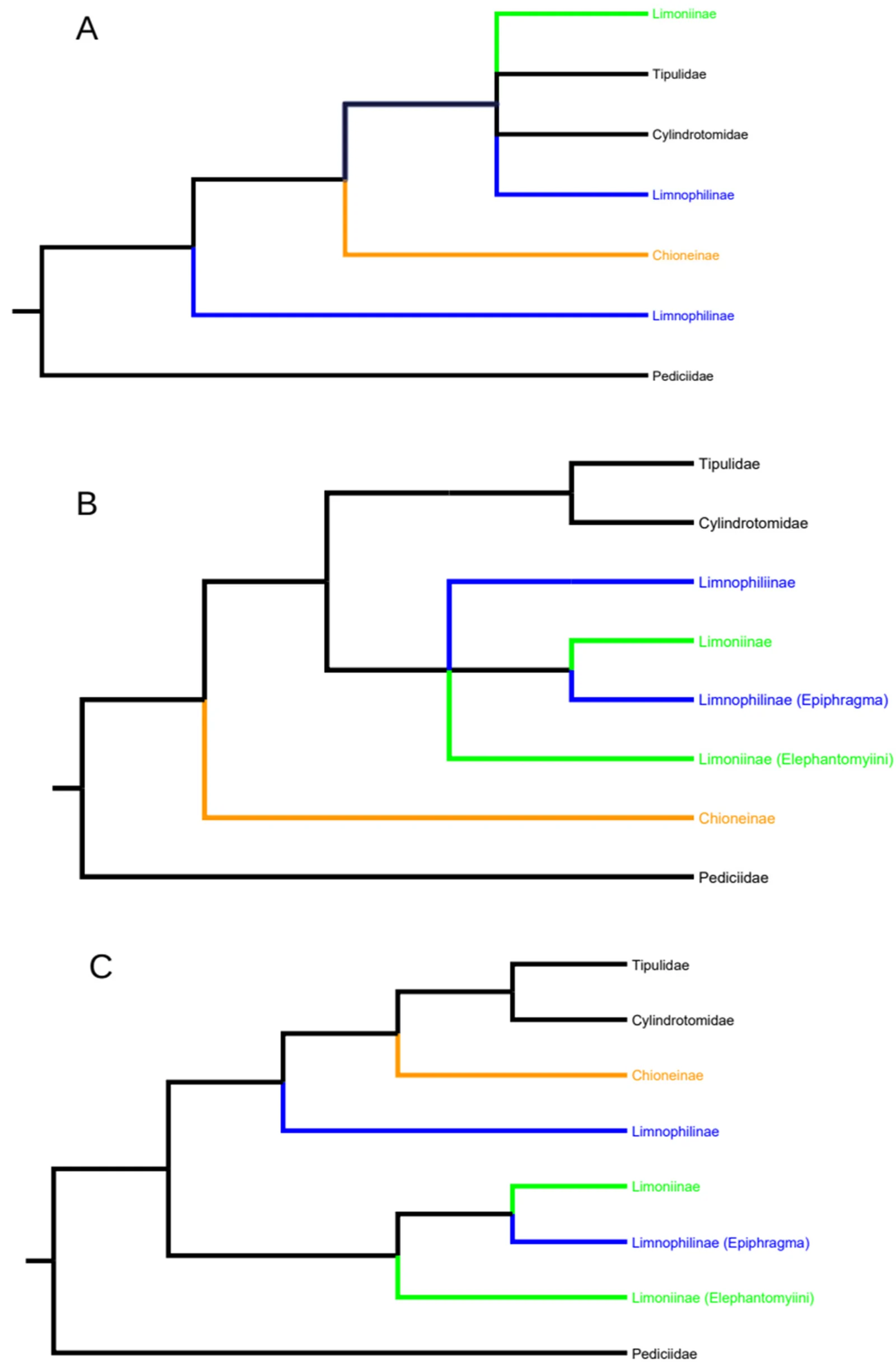
Examples of molecular phylogenies of Limoniidae. (**A**)—modified from the Bayesian inference analysis of the concatenated dataset of Ahonen [29]. (**B**)—modified from the Bayesian inference analysis of the mitochondrial genome dataset containing the first and second codon positions of the protein-coding genes plus the RNA genes of Kang et al. [34]. (**C**)—modified from the Bayesian inference analysis based only on the mitochondrial genome protein-coding genes of Kang et al. [34].

**Figure 6:**
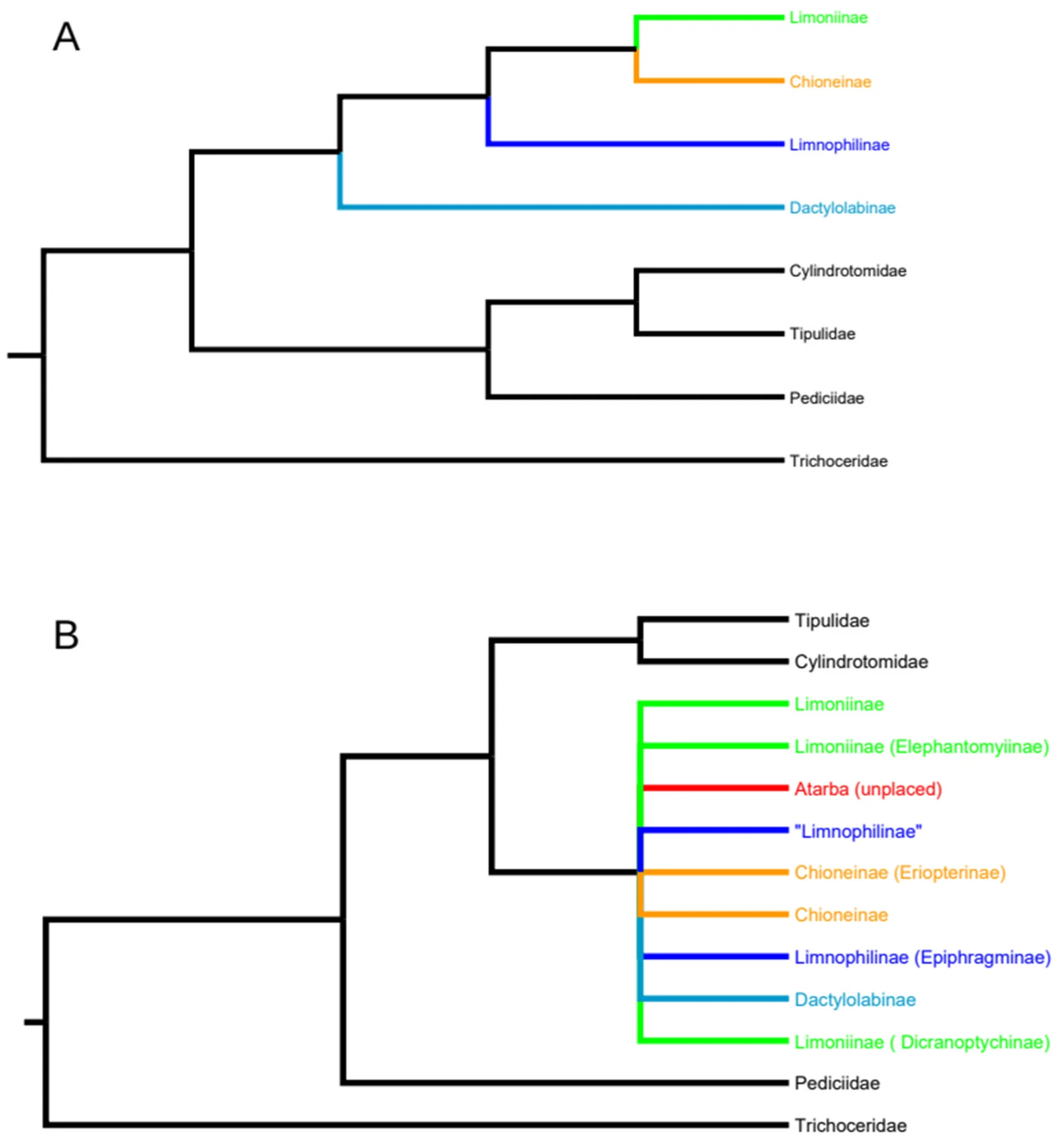
Comparison between the “traditional classification” of Tipulomorpha proposed by Starý and a molecular phylogeny-based classification. (**A**)—Qualitative morphological phylogeny of Tipulomorpha according to Starý [4]. (**B**)—Classification proposed by Petersen et al. [5]. Note that the classification proposed by Starý [4] remains the one most widely used by crane fly taxonomists. However, this classification is not supported by studies employing either morphological or molecular approaches. The basal position of Pediciidae is present in both schemes. Remarkable differences include the position of the Cylindrotomyiidae–Tipulidae sister clade, which is placed basally in Starý [4] but represents the most derived clade in molecular studies, as well as the non-monophyly of Limoniidae.

The first molecular study targeting relationships within Tipuloidea was conducted by Ahonen [29], who examined subfamily-level relationships across all four crane fly families (Figure 5A). Sampling was incomplete, as no representatives of the Tipulidae subfamilies Ctenophorinae and Dolichopezinae or the Limoniidae subfamily Dactylolabinae were included. The analysis used the mitochondrial gene cytochrome c oxidase subunit I (COI) and the nuclear gene elongation factor 1-alpha (EF-1α), with 654 bp and 950 bp respectively successfully aligned across 36–37 species. Trees inferred using Maximum Parsimony, Neighbor Joining, Maximum Likelihood, and Bayesian inference showed poor resolution for individual genes, but the concatenated dataset produced more stable topologies [29]. Pediciidae emerged as the basal lineage of Tipuloidea. Limoniidae, however, appeared paraphyletic, indicating that the group, as currently defined, does not include all descendants of a common ancestor and likely represents a grade of early-diverging lineages within Tipuloidea rather than a natural, monophyletic group. Chioneinae and Limoniinae were inferred as monophyletic, while Limnophilinae was not. Despite limited sampling, 37 species, all from Finland, the study supported earlier hypotheses such as the close relationship of Cylindrotomidae and Tipulidae and the paraphyly of Limnophilinae [14]. Petersen et al. [5] combined morphological and molecular data to further investigate Tipuloidea phylogeny. Their dataset included 45 species. Their analysis focused on the nuclear genes 28S rRNA, CAD (with third codon positions included and excluded), and 100 morphological characters from larval, pupal, and adult stages. Phylogenetic trees were inferred using Maximum Parsinomy and Bayesian inference. Analyses were run on individual gene partitions, morphological data alone, and a combined dataset. Bayesian Inference was applied only to the combined dataset. Single-partition analyses produced conflicting topologies, while the combined dataset yielded more robust and congruent trees. Their results strongly supported the monophyly of Tipuloidea, with Pediciidae as the most basal lineage, sister to all other crane flies. A well-supported clade comprising Tipulidae and Cylindrotomidae was also recovered, and Limoniidae were again retrieved as paraphyletic. Not all branches were well-resolved; for instance, the genus *Atarba* Osten Sacken, 1869, could not be confidently assigned to any clade (Table 4; Figure 6B). Based on these findings, Petersen et al. [5] proposed a revised two-family classification of crane flies, comprising Pediciidae and Tipulidae. In this framework, Limoniidae are divided into at least eight subfamilies nested within Tipulidae, alongside Cylindrotominae and Tipulinae (Table 4). However, as the authors acknowledged, their sampling included fewer than 10% of extant crane flies species, and additional studies are necessary to confirm or refute this proposed classification scheme.

In recent years, a group of Chinese researchers has sequenced the complete mitochondrial genomes of several Limoniidae species and examined phylogenetic relationships among crane flies’ lineages, although the number of species included was limited [30,31,32,33,34,35].

Kang et al. [30] sequenced and characterized the complete mitochondrial genome of *Chionea crassipes gracilistyla* Alexander, 1936 and reconstructed the phylogeny of Tipuloidea using Bayesian inference. Their dataset included four Limoniidae species, one Pediciidae, one Cylindrotomidae, and two Tipulidae, with two Trichoceridae species used as outgroup. The resulting phylogeny recovered Pediciidae as the earliest-diverging lineage within Tipuloidea, supported a sister-group relationship between Cylindrotomidae and Tipulidae, and suggested that Limoniidae is not monophyletic. Similarly, Ren et al. [31,32,33] sequenced and characterized the complete mitochondrial genomes of *Limonia phragmitidis* (Schrank, 1781), *Nephrotoma tenuipes* (Riedel, 1910), and *Pseudolimnophila brunneinota* Alexander, 1913. Phylogenetic analyses were also conducted using Bayesian inference. The first study included four Limoniidae species, one Pediciidae, one Cylindrotomidae, and two Tipulidae species, with a Trichoceridae species as the outgroup [31].

The latter two studies expanded taxon sampling to four Limoniidae species [32] or five Limoniidae species [33], one Pediciidae, one Cylindrotomidae, and four Tipulidae species, using two Trichoceridae species and one Anisopodidae species as outgroup [32,33]. Despite these differences in taxon sampling, all analyses consistently recovered Pediciidae as the sister lineage to the remaining Tipuloidea, supported a sister-group relationship between Cylindrotomidae and Tipulidae, and placed Limoniidae as the sister group to the Cylindrotomidae–Tipulidae clade. However, the latter family was consistently recovered as non-monophyletic.

Kang et al. [34] sequenced the mitochondrial genome of two species of the tribe *Elephantomyiini*, *Elephantomyia inulta* Alexander, 1938 and *Helius pluto* Alexander, 1932 representing the first characterization of the mitochondrial genome of this tribe. In addition, they build a phylogenetic tree based on both Bayesian inference and Maximum Likelihood. They used 12 Limoniidae species, one Pediciidae species, one Cylindrotomidae and 13 species of Tipulidae with three Trichoceridae species used as outgroup. The authors analyzed four different mitochondrial genome datasets to infer the phylogeny of Tipuloidea.

Their results indicated that the Pediciidae were the most basal lineage of the Tipuloidea, reaffirmed the sister relationship between the Cylindrotomyiidae and the Tipulidae and reported the non-monophyly of the Limoniidae (Figure 5B,C).

In contrast, Xu et al. [35] focused on the tribe *Dicranoptychini* in China. In addition to describing two species new to science and reporting a new country record for China, the authors provided an identification key to the Chinese species of *Dicranoptycha* Osten Sacken, 1860 and sequenced the complete mitochondrial genome of the newly described species *Dicranoptycha shandongensis* Xu, Chen & Zhang, 2023. They also reconstructed the phylogeny of Tipuloidea using both Bayesian inference and Maximum Likelihood. Their dataset included 11 Limoniidae species, one Cylindrotomidae species, and three Tipulidae species. Unlike previous studies, they used a single Pediciidae species as the outgroup because earlier analyses had consistently recovered Pediciidae as the earliest-diverging lineage within Tipuloidea. Their results were largely consistent with previous studies, recovering Limoniidae as a non-monophyletic group and supporting a sister-group relationship between Cylindrotomidae and Tipulidae.

## 3. Improving the Classification of Limoniidae

Only a limited number of studies have applied molecular approaches to test or refine the classification of Limoniidae, and these investigations have generally been constrained by both sparse taxon sampling and the use of few genetic markers.

Analyses based on single genes have frequently produced highly incongruent phylogenetic trees [5,19] whereas better-resolved and more stable topologies have been obtained when multiple loci were concatenated [18,25] or when molecular and morphological data were analysed jointly [5]. These findings are consistent with studies reported in the literature which show that concatenating sequences produces better-resolved topologies [36,37]. However, it is well established that relying on a limited number of molecular markers increases the chance of stochastic error in phylogenetic inference. Stochastic error arises because only a finite number of informative characters are sampled, allowing random similarity and homoplasy to obscure true evolutionary relationships [38]. As a result, phylogenetic reconstructions based on a small number of nucleotide or amino acid sites may recover relationships that reflect chance rather than shared ancestry. Furthermore, genetic variation does not necessarily reflect adaptive evolution but may instead result from neutral evolutionary processes such as genetic drift, gene duplication, or gene loss. Consequently, single-gene or multi-locus datasets often contain a limited amount of phylogenetically informative signal, reducing the accuracy and robustness of inferred evolutionary relationships [39]. Since the mid-2000s, advances in high-throughput sequencing technologies have enabled the generation of genome-scale molecular datasets, giving rise to the field of phylogenomics. Phylogenomics integrates evolutionary biology and genomics by using genome-scale data to reconstruct species’ evolutionary histories, investigate gene family evolution, predict gene functions, and infer complex evolutionary events such as horizontal gene transfer. In addition, comparative genomic approaches exploit genome-wide information to reconstruct species trees [40]. The application of genome-scale data to phylogenetic reconstruction was initially met with considerable enthusiasm, with some researchers suggesting that phylogenomics would resolve the long-standing problem of phylogenetic incongruence [41]. The rationale was that increasing the amount of sequence data would reduce stochastic error and provide sufficient phylogenetic signal to recover the evolutionary history of organisms. It was even advocated that increasing the number of genetic markers alone would be sufficient to recover stable topologies [42,43]. Subsequent work, however, has demonstrated that increasing taxon sampling can be equally or even more important for improving phylogenetic inference [44,45], provided that taxa are selected strategically rather than at random [46,47].

Since its introduction, phylogenomics has substantially improved our understanding of major evolutionary radiations, including placental mammals [48,49], land plants [50], and insects [51]. Nevertheless, important uncertainties remain. While the large number of characters used in phylogenomics datasets dramatically reduces stochastic error associated with limited sampling, uncertainties inherent to the data and, in particular, to the choice and adequacy of evolutionary models continue to pose significant challenges [52]. These sources of bias are collectively referred to as systematic errors.

Common systematic errors in phylogenomics include compositional bias, long-branch attraction, model misspecification, and heterotachy [53,54]. Compositional bias arises when distantly related taxa are incorrectly grouped because their sequences share similar nucleotide or amino acid compositions rather than true common ancestry. Long-branch attraction occurs when rapidly evolving lineages are erroneously inferred as closely related, due to the accumulation of convergent or homoplastic substitutions [55]. The choice of an evolutionary model is a critical step in phylogenetic inference [56]. All models necessarily simplify biological reality, and some violations of their assumptions are unavoidable. However, when a model deviates substantially from the “true” evolutionary process, it can lead to incorrect tree topologies, a phenomenon known as model misspecification. Heterotachy refers to changes in evolutionary rates at the same site or locus over time, such that different lineages experience different rate regimes during their evolutionary history [53]. Many commonly used models assume stationarity and homogeneity of evolutionary rates across taxa and can bias phylogenetic inference.

An additional source of incongruence is gene tree discordance due to processes such as incomplete lineage sorting [57,58,59].

However, given the remarkable species richness of the family Limoniidae and the incongruent phylogenetic hypotheses recovered by previous molecular studies, phylogenomic approaches appear to be the most promising strategy for resolving the evolutionary relationships within the family.

Limoniidae, together with other crane fly lineages, have a long tradition of taxonomic and morphological study. However, some biogeographical regions remain poorly explored [2], and molecular data remain unavailable for most species, particularly at the genomic scale. Therefore, targeted morphological analyses may play a critical role in guiding molecular sampling by identifying key taxa whose inclusion is likely to maximize phylogenetic signal. A major gap in our understanding of Limoniidae systematics concerns the limited knowledge of larval morphology: only about one-third of Limoniidae larvae have been described to date [2,14]. Detailed studies of larval stages can yield crucial phylogenetic information. For instance, Podėnienė & Gelhaus [60] transferred the genus *Phyllolabis* Osten Sacken, 1877 from the subfamily Chioneinae back to Limnophilinae based on larval characters of *Phyllolabis mongolica* Podėnienė & Gelhaus, 2012.

Taken together, these considerations suggest that dense taxon sampling is essential given the many uncertainties surrounding the current classification of Limoniidae. Comprehensive taxon sampling also facilitates the detection of systematic errors arising from lineage-specific differences in sequence composition and other sources of compositional bias [61]. Despite their important ecological roles [62], Limoniidae and other crane flies are generally considered to be of limited economic importance and, consequently, remain relatively understudied. Consequently, securing funding for large-scale phylogenetic and phylogenomic research on these flies may prove challenging. Given the exceptional diversity of the family and the scale of the work required, the most effective way forward may be through coordinated efforts among research groups specialized in crane fly systematics. Such collaborations would facilitate broad taxon sampling, the integration of complementary expertise, and the generation of the genomic resources needed.

Considering the complexity of such an endeavour, genome skimming may represent a practical compromise, providing genome-scale data at a relatively low cost while enabling the reconstruction of robust phylogenetic hypotheses [63,64]. Moreover, genome skimming is particularly well suited to the analysis of museum specimens [65,66,67], allowing the vast collections housed in natural history museums to be incorporated into phylogenomic studies and substantially increasing taxon sampling. Natural history museum collections contain numerous underutilized specimens that could provide invaluable material for such projects [68].

## 4. Concluding Remarks

Accurate biological classification is a fundamental objective in systematics, providing a framework for understanding evolutionary relationships, recognizing and naming species, and interpreting patterns of adaptation, insights that are often essential for effective conservation strategies. The Limoniidae, a large and highly diverse lineage, have proven particularly challenging to classify. Most morphological and molecular studies have focused on two broad questions: (1) the phylogenetic relationships of Limoniidae with the other crane fly families, including the relationship between Tipuloidea and Trichoceridae and the position of crane flies within the lower Diptera; and (2) the evolutionary relationships among the subfamilies of Limoniidae and between these subfamilies and other crane fly lineages. Only a limited number of studies have addressed these questions using molecular data, and none has provided a definitive resolution of the phylogenetic relationships within Limoniidae or of the placement of Tipuloidea within Diptera. Nevertheless, several consistent patterns have emerged. The Limoniidae and other crane fly lineages appear to form a monophyletic superfamily, Tipuloidea. As originally hypothesized by Hennig [6], Tipuloidea is the sister group to Trichoceridae, and together they constitute the infraorder Tipulomorpha. The phylogenetic position of Tipulomorpha within Diptera, however, remains unresolved. Studies have recovered Tipulomorpha either as one of the earliest-diverging dipteran lineages (Figure 4) or as occupying an intermediate position within the dipteran phylogeny (Figure 3B). Interestingly, analyses incorporating broader taxon sampling across Diptera tend to recover Tipulomorpha as an early-diverging lineage [18,28], whereas studies based on more limited taxon sampling generally place it in a more intermediate position [26,27]. None of the studies examined that used molecular approaches recovered Tipulomorpha as a highly derived lineage, in contrast to the hypothesis proposed by Oosterbroek and Courtney [24]. Studies addressing the internal relationships of Limoniidae have been considerably more difficult to resolve, highlighting the current uncertainty surrounding the classification of the family. The studies reviewed recovered contrasting hypotheses (Figure 6). In Ahonen [29], Bayesian inference recovered Limnophilinae as the earliest-diverging lineage within Limoniidae. Limoniinae was inferred to be the most derived subfamily, branching after the divergence of both Tipulidae and Cylindrotomidae. In the revised classification proposed by Petersen et al. [5] (Figure 6B) the earliest-diverging lineage within Limoniidae was represented by the genus *Dicranoptycha*, traditionally assigned to Limoniinae, and *Dactylolabis* which Starý [4] had previously placed in the subfamily Dactylolabinae. Limnophilinae was divided into two distinct clades: one represented by *Epiphragma* Osten Sacken, 1860 as an early-diverging lineage and a second occupying an intermediate position within the family. The authors noted that denser taxon sampling might further subdivide this latter clade. Chioneinae was also recovered as two separate clades, whereas part of Limoniinae constituted the most derived lineage within the family. The genus *Atarba*, placed in the subfamily Chioneinae [1], could not be placed (Figure 6B). The mitochondrial genome studies of Kang et al. [30] and Ren et al. [31,32,33], included only four or five Limoniidae species. Ren et al. [31,32,33] consistently recovered *Limonia* and *Rhipidia* Meigen, 1818 as sister taxa. However, the limited taxon sampling prevented robust conclusions regarding deeper relationships within the family. Kang et al. [34] expanded the sampling to 12 Limoniidae species but obtained conflicting results depending on the dataset analysed (Figure 5B,C). Interestingly, the genera *Elephantomyia* and *Helius* formed a clade, a result consistent with Petersen et al. [5].

In addition, the sister-group relationship between *Limonia* and *Rhipidia* recovered in previous mitochondrial studies [31,32,33] was no longer supported, suggesting that increased taxon sampling within Limoniinae altered the inferred relationships [34]. Xu et al. [35] analysed several combinations of mitochondrial datasets and likewise recovered inconsistent topologies. *Symplecta hybrida*, a Chioneinae species, was consistently retrieved as the earliest-diverging representative of the Limoniidae. Limnophilinae was divided into two or three clades, whereas Limoniinae was split into two clades. Notably, *Dicranoptycha* was recovered outside the remaining Limoniidae, a result similar to that proposed by Petersen et al. [5]. Furthermore, *Dicranoptycha* consistently formed a clade with *Epiphragma*. However, the *Epiphragma–Dicranoptycha* clade occupied an intermediate position in three of the four analyses, differing from both Petersen et al. [5] and Kang et al. [34]. Taken together, these studies reveal a highly inconsistent picture of relationships within Limoniidae.

At least part of this inconsistency is likely attributable to differences in taxon sampling, gene selection, dataset composition, and analytical approaches among the studies reviewed.

Nonetheless, as observed in studies addressing the phylogeny of Tipuloidea, some consistent patterns have emerged at higher taxonomic levels. Within Tipuloidea, Pediciidae likely represent the earliest-diverging lineage, while Tipulidae and Cylindrotomidae consistently emerge as sister groups. In contrast, the monophyly of Limoniidae has not been supported by most studies, suggesting that the family is paraphyletic or possibly even polyphyletic. The only explicit alternative classification framework proposed to date [5] advocates a two-family system comprising Pediciidae and Tipulidae (Table 4; Figure 6B). Under this scheme, Limoniidae sensu Starý [4] (Figure 6A) would be subdivided into multiple smaller subfamilies and subsumed within Tipulidae. However, the phylogenetic hypothesis advanced by Petersen et al. [5] is likely constrained by limited taxon sampling, as evidenced by several unresolved clades (Table 4; Figure 6B). In conclusion, the classification proposed by Starý in 1992 [4] (Figure 6A) is not supported by studies employing molecular approaches. Resolving the phylogeny of Limoniidae will likely require an integrative approach that combines morphological data, dense taxon sampling, and phylogenomic analyses. Achieving this goal will depend on coordinated collaboration among specialists, the strategic application of cost-effective sequencing approaches such as genome skimming, and the incorporation of specimens from natural history museum collections. Given the limited number of taxonomists specializing in Limoniidae and other crane flies, a practical strategy may be to adopt a stepwise phylogenomic framework that progressively expands taxon sampling and reassesses phylogenetic relationships as new taxa are incorporated. Together, these approaches provide the most promising framework for resolving the classification and evolutionary relationships of Limoniidae.

## Figures and Tables

**Table 4:** Provisonial classification proposed by Petersen et al. [5]. In this framework two families of crane flies are retained: Pediciidae and the Tipulidae. The former Limoniidae are splitted in at least eight subfamilies and incorporated in the Tipulidae. The former families Tipulidae and Cylindrotomidae are treated as subfamilies whitin Tipulidae. New subfamilies proposed by Petersen et al. [5] are highlighted in yellow. The genus *Atarba*, shown in red, could not be placed in any subfamily. The Limnophilinae, highlighted in grey, likely represent a non-monophyletic assemblage, and additional data are required to achieve a stable classification of this group.

Classification Proposed by Petersen et al. [5]	
Family	Subfamilies
Pediciidae	
Tipulidae	Dicranoptychinae
	Dactylolabinae
	Epiphragminae
	Chioneinae
	Eriopterinae
	“Limnophilinae”
	*Atarba*?
	Elephantomyiinae
	Limoniinae
	Cylindrotominae
	Tipulinae

## Data Availability

All data and information’s are embedded in the text.
